# Laparoscopic vs open colorectal cancer surgery in elderly patients: short- and long-term outcomes and predictors for overall and disease-free survival

**DOI:** 10.1186/s12893-019-0596-3

**Published:** 2019-09-14

**Authors:** Sicheng Zhou, Xuewei Wang, Chuanduo Zhao, Qian Liu, Haitao Zhou, Zhaoxu Zheng, Zhixiang Zhou, Xishan Wang, Jianwei Liang

**Affiliations:** Department of Colorectal Surgery, National Cancer Center/National Clinical Research Center for Cancer/Cancer Hospital, Chinese Academy of Medical Sciences and Peking Union Medical College, Beijing, 100021 China

**Keywords:** Colorectal cancer, Open surgery, Laparoscopic surgery, Surgical outcomes, Elderly patients

## Abstract

**Background:**

Colorectal cancer is common in elderly patients. Laparoscopy is widely used to approach this kind of disease. This study was to examine short-term outcomes and long-term survival for laparoscopic and open surgery in elderly patients with colorectal cancer.

**Methods:**

From January 2007 to December 2018, patients with colorectal cancer older than 80 operated at China National Cancer Center were included in the study. Propensity score matching (PSM) was used to minimize the adverse effects. The clinical data between open and laparoscopic surgery was compared, and the effect of factors on overall survival (OS) and disease-free survival (DFS) was analyzed by Cox proportional hazard model.

**Results:**

Ninety-three pairs were selected after PSM. Patients in laparoscopic group had less intraoperative blood loss, postoperative complications, time to first flatus, time to oral feeding, postoperative hospital stay, and higher retrieved lymph node (*P* < 0.05). The OS and DFS rates were similar (*P* > 0.05), besides the CEA level, III/IV stage, and perineural invasion were independent predictors of survival (*P* < 0.05).

**Conclusion:**

In elderly patients with colorectal cancer, laparoscopic surgery had better short-term outcomes than open surgery. CEA level, III/IV stage, and perineural invasion were reliable predictors for OS and DFS.

## Introduction

According to the Globocan 2012 database of the World Cancer Research Centre, the incidence of colorectal cancer in those over 75 years old in China is approximately 78,200 every year and it accounted for 18.08% of the global incidence [1]. Laparoscopic technique for colorectal cancer has been widely applied in clinical practice due to its advantages of small incision, quick recovery, and shorter hospitalization compared with open surgery [2–4]. Meanwhile, its short-term results and long-term efficacy have been confirmed by a series of a large sample, multicenter randomized controlled studies [5–8]. However, there are few literature on whether elderly patients with colorectal cancer can also benefit from laparoscopic surgery [9–12]. Therefore, we designed a single-center and propensity score-matched analysis to investigate the short-term outcomes and survival rates of laparoscopic and open colorectal surgery, as well as the reliable predictors for OS and DFS in elderly patients with colorectal cancer.

## Material and methods

### Patients

After approved by the ethics committee of Cancer Hospital, Chinese Academy of Medical Sciences (NCC 2017-YZ-026, Oct 17, 2017), all curatively operated colorectal cancer patients were collected between January 2007 and December 2018 at National Cancer Centre/National Clinical Research Centre for Cancer/Cancer Hospital, Chinese Academy of Medical Sciences and Peking Union Medical College. The inclusion criteria included: [1] older than 80 years old; [2] treated by laparoscopic or open surgery for colorectal cancer. The exclusion criteria were as follows: [1] emergency surgery for reasons such as intestinal obstruction, bleeding or perforation; [2] double primary cancers; [3] treated by palliative surgery; [4] treated by neoadjuvant therapy before surgery. All patients were treated by colorectal surgeons with more than 20 years of surgical experience, and all enrolled patients underwent radical surgery. Besides, written informed consent was obtained from each patient included in the study.

### Clinical data collection

In this study, clinical data were collected based on electronic records and included age, gender, body mass index (BMI), preoperative hemoglobin (HGB), preoperative albumin, American Society of Anesthesiologists (ASA) score, comorbidities, previous abdominal surgery, tumor location, tumor differentiation, and tumor nodes metastasis (TNM) stage. Besides, the perioperative outcomes were also collected including the surgical outcomes, pathological outcomes, and the postoperative recovery. The surgical outcomes included the duration of operation, intraoperative blood loss, blood transfusion, intraoperative complication, postoperative complication, mortality, and intensive care unit (ICU) stay. The pathological outcomes included the retrieved lymph node, tumor size, perineural invasion, vascular invasion, and positive circumferential resection margin (CRM). Postoperative recovery included time to first flatus, time to oral feeding, and postoperative hospital days. The parameters of the postoperative recovery were all calculated based on the end of the operation. Postoperative complications were defined according to the Clavien­Dindo classification [13], including wound infection, anastomosis leakage, ileus, urosepsis, pneumonia, pelvic abscess, arrhythmia, pleural effusion, delayed gastric emptying, and bacteremia.

### Survival outcomes collection

After surgery, all patients received a follow-up survey every 3–6 months in the first 3 years by outpatient visits. In these 3 years, the patients were diagnosed by physical and laboratory examinations including biomarkers (CEA and CA-199) at each visit, CT scans of the chest, abdomen, and pelvis at every half year, and complete colonoscopy at each year. After the first 3 years, the patients received follow-up survey every 6–12 months by outpatient visits or telephone until the death of the patients due to recurrence and metastasis of colorectal cancer or December 31, 2018. Based on this follow-up survey, the data about the 5-year overall survival (OS) and disease-free survival (DFS) were collected.

### Statistical analysis

The SPSS

.0 for Windows (IBM Corp, Armonk, NY, USA) was used for data analysis in this study. According to the operation type, patients were divided into laparoscopy and open group. Quantitative data were analyzed by the Mann-Whitney U test and presented as mean ± standard deviation ($\overline{x}\pm SD$). Categorical data were analyzed by the Chi-squared test and presented as frequency and percentage. Propensity score matching (PSM) was carried out by logistic regression to reduce the effects of selection bias in these 2 groups. The matching ratio was 1:1, and the covariates included age, gender, BMI, preoperative HGB level, preoperative albumin level, ASA class, comorbidity, previous abdominal surgery, tumor location, tumor differentiation, and TNM stage. The Kaplan-Meier method was performed to calculate the survivals of the patients treated with different operation types in the 2 groups, and the differences of the survival outcomes (OS and DFS) were compared by a log-rank test. The statistically significant variables in univariate analysis were subsequently tested by multivariate analysis through a Cox-regression model, and the effect of each variable was assessed by the Hazard ratio (HR) and 95% confidence interval (95% CI). All tests were two-sided, and the *P* value less than 0.05 was considered to indicate statistical significance.

## Results

### Clinical and pathological characteristics

Three-hundreds and 13 patients were enrolled in the study. Among them, 93 marched pairs were selected through propensity scoring. The clinical and pathological characteristics before and post matching groups were as shown in Table 1. Before matching, there were significant differences in aspects of BMI, preoperative HGB, preoperative albumin, ASA, comorbidity, tumor location, differentiation (*P* < 0.05) between the open group and laparoscopic group.

**Table 1:** Clinical and pathological characteristics of elderly patients with colorectal cancer before and after matching

Variables	Total cohort			Matched cohort		
	OPEN (*n* = 143)	LAP (*n* = 170)	*P*	OPEN (*n* = 93)	LAP (*n* = 93)	*P*
Age (years, mean ± SD) (range)	82.3 ± 2.1 (80–92)	82 ± 2.1 (80–94)	0.280	82.1 ± 2.1 (80–92)	82.0 ± 2.2 (80–94)	0.814
Gender			0.445			0.655
Male	91 (63.6%)	101 (59.4%)		56 (60.2%)	53 (57%)	
Female	52 (36.4%)	69 (40.6%)		37 (39.8%)	40 (43%)	
BMI (kg/m^2^, mean ± SD) (range)	22.7 ± 3.3 (15.8–33.8)	23.7 ± 3.3 (15.6–34.1)	0.009	23.4 ± 3.0 (15.8–33.8)	23.2 ± 3.3 (16.8–34.1)	0.703
Preoperative HGB (g/L, mean ± SD) (range)	120.0 ± 19.1 (49–165)	124.5 ± 18.7 (62–161)	0.045	120.2 ± 19.6 (49–165)	121.5 ± 19.2 (62–161)	0.645
Preoperative albumin (g/L, mean ± SD) (range)	37.2 ± 4.3 (26.8–46.2)	40.2 ± 4.1 (29.1–46.8)	< 0.001	38.4 ± 4.0 (29.5–46.2)	38.9 ± 4.0 (29.1–46.2)	0.409
ASA score			< 0.001			0.378
I-II	52 (36.4%)	111 (65.3%)		47 (50.5%)	53 (57%)	
III-IV	91 (63.6%)	59 (34.7%)		46 (49.5%)	40 (43%)	
Comorbidity			0.008			0.761
Yes	83 (58.0%)	123 (72.4%)		60 (64.5%)	58 (62.4%)	
No	60 (42.0%)	47 (27.6%)		33 (35.5%)	35 (37.6%)	
Previous abdominal surgery			0.456			0.721
Yes	30 (21.0%)	30 (17.6%)		19 (20.4%)	21 (22.6%)	
No	113 (79.0)	140 (82.4)		74 (79.6)	72 (77.4)	
Tumor location			0.007			0.698
Right colon	51 (35.7%)	39 (23%)		32 (34.4%)	31 (33.3%)	
Left colon	10 (6.9%)	4 (2.3%)		7 (7.5%)	4 (4.3%)	
Sigmoid colon	31 (21.7%)	42 (24.7%)		21 (22.6%)	19 (20.4%)	
Rectum	51 (35.7%)	85 (50%)		33 (35.5%)	39 (42%)	
Tumor differentiation			0.029			0.895
Poor	29 (20.3%)	45 (26.5%)		23 (24.7%)	24 (25.8%)	
Median	105 (73.4%)	123 (72.4%)		67 (72%)	67 (72%)	
High	9 (6.3%)	2 (1.1%)		3 (3.3%)	2 (2.1%)	
TNM stage*			0.213			0.860
I	8 (5.6%)	12 (7.1%)		8 (8.6%)	10 (10.8%)	
II	45 (31.5%)	43 (25.3%)		30 (32.2%)	27 (29.0%)	
III	83 (58.0%)	112 (65.8%)		53 (57.0%)	55 (59.1%)	
IV	7 (4.9%)	3 (1.8%)		2 (2.2%)	1 (1.1%)	
Preoperative CEA (ng/mL)						0.503
≤ 5	102 (71.3%)	133 (78.2%)	0.159	67 (72%)	71 (76.3%)	
> 5	41 (28.7%)	37 (21.8%)		26 (28%)	22 (23.7%)	

*OPEN* open surgery; *LAP* laparoscopic surgery; *BMI* body mex index; *HGB* hemoglobin; *ASA* American Society of Anesthesiologists; *TNM* tumor nodes metastasis; *CEA* carcinoembryonic antigen; *SD* standard deviation; * evaluated based on 7th edition of AJCC

Before matching, the open group had significantly lower BMI (22.7 ± 3.3 kg/m^2^ vs. 23.7 ± 3.3 kg/m^2^, *P* = 0.009), preoperative HGB (120.0 ± 19.1 g/L vs. 124.5 ± 18.7 g/L, *P* = 0.045), and preoperative albumin (37.2 ± 4.3 g/L vs. 40.2 ± 4.1 g/L, *P* = 0.045), significantly higher preoperative ASA scores (63.6% vs. 34.7% ASA III-IV, *P* < 0.001), and less comorbidities than the laparoscopic group (58.0% vs. 72.4%, *P* = 0.008). The primary tumor localization was more frequent in the right and left colon and less frequent in the sigmoid colon and rectum in the open group (*P* = 0.007). There were more patients with poor tumor differentiation in the laparoscopic group than the open group (26.5% vs. 20.3%, *P* = 0.029).

After matching, the laparoscopic group and open group were well balanced in aspects of age, gender, BMI, preoperative HGB, preoperative albumin, ASA score, comorbidity, previous abdominal surgery, tumor location, tumor differentiation, TNM stage, and preoperative CEA.

### Short-term outcomes

The short-term outcomes, including the surgical outcomes, pathological outcomes, and postoperative recovery, in matched cohorts were as shown in Table 2. There was a significant difference in aspects of intraoperative blood loss and postoperative complication between the two groups. The laparoscopic group had significantly lower intraoperative blood loss (50.9 ± 44.9 mL vs. 108.1 ± 78.5 mL, *P* < 0.001) and lower occurrence of postoperative complication (10.8% vs. 26.9%, *P* = 0.005). According to the Clavien Dindo classification, the incidence of grade I-II complications in the laparoscopic group was significantly higher (17.2% vs. 6.5%, *P* = 0.023). The most common morbidity in the open group was wound infection in 9 patients (9.7%), followed by ileus in 5 patients (5.4%), anastomosis leakage in 4 patients (4.3%), and delayed gastric emptying in 4 patients (4.3%). In the laparoscopic group, the most common morbidities were anastomosis leakage in 2 patients (2.2%), ileus in 2 patients (2.2%) and pneumonia in 2 patients (2.2%). No patient died during the operation. For the pathological outcome, the retrieved lymph node was significantly higher in the laparoscopic group (20.3 ± 10.5 day**s** vs. 17.2 ± 9.1 day**s**, *P* = 0.030). Time to first flatus (4.5 ± 1.6 day**s** vs. 5.5 ± 2.1 day**s**, *P* = 0.001), time to oral feeding (4.8 ± 2.2 day**s** vs. 5.9 ± 2.5 day**s**, *P* = 0.003), and postoperative hospital stay (9.6 ± 3.3 days vs. 12.2 ± 5.5 day**s**, *P* < 0.001) were all significantly lower in the laparoscopic group.

**Table 2:** Perioperative outcomes of 186 elderly patients with colorectal cancer in matched cohorts

Variables	OPEN (*n* = 93)	LAP (*n* = 93)	*P*
Surgical outcome			
Duration of operation (min, mean ± SD) (range)	149.1 ± 53.9 (45–350)	161.2 ± 55.3 (65–380)	0.064
Intraoperative blood loss (mL, mean ± SD) (range)	108.1 ± 78.5 (10–400)	50.9 ± 44.9 (10–200)	< 0.001
Blood transfusion	21 (22.6%)	15 (16.1%)	0.265
Intraoperative complication	0 (0%)	1 (1.1%)	1.000
Postoperative complication	25 (26.9%)	10 (10.8%)	0.005
Wound infection	9 (9.7%)	1 (1.1%)	0.009
Anastomosis leakage	4 (4.3%)	2 (2.2%)	0.678
Ileus	5 (5.4%)	2 (2.2%)	0.441
Uroschesis	1 (1.1%)	1 (1.1%)	1.000
Pneumonia	3 (3.2%)	2 (2.2%)	1.000
Pelvic abscess	0 (0%)	1 (1.1%)	1.000
Arrhythmia	3 (3.2%)	1 (1.1%)	1.000
Pleural effusion	2 (2.1%)	0 (0%)	0.477
Delayed gastric emptying	4 (4.3%)	1 (1.1%)	0.365
Bacteremia	1 (1.1%)	0 (0%)	1.000
Postoperative complication (Clavien­Dindo classification)			
I-II	16 (17.2%)	6 (6.5%)	0.023
III-V	9 (9.7%)	4 (4.3%)	0.150
Mortality	0 (0%)	0 (0%)	–
ICU staying	13 (13.9%)	7 (7.5%)	0.156
Pathological outcome			
Retrieved lymph node (mean ± SD) (range)	17.2 ± 9.1 (1–55)	20.3 ± 10.5 (5–58)	0.030
Tumor size			0.378
< 5 cm	46 (49.5%)	52 (55.9%)	
≥ 5 cm	47 (50.5%)	41 (44.1%)	
Perineural invasion	22 (23.7%)	18 (19.4%)	0.475
Vascular invasion	28 (30.1%)	24 (25.8%)	0.513
Positive CRM	1 (1.1%)	0 (0%)	0.316
Postoperative recovery			

*OP* open surgery; *LAP* laparoscopic surgery; *CRM* circumferential resection margin

### Survival analysis

**Fig. 1:**
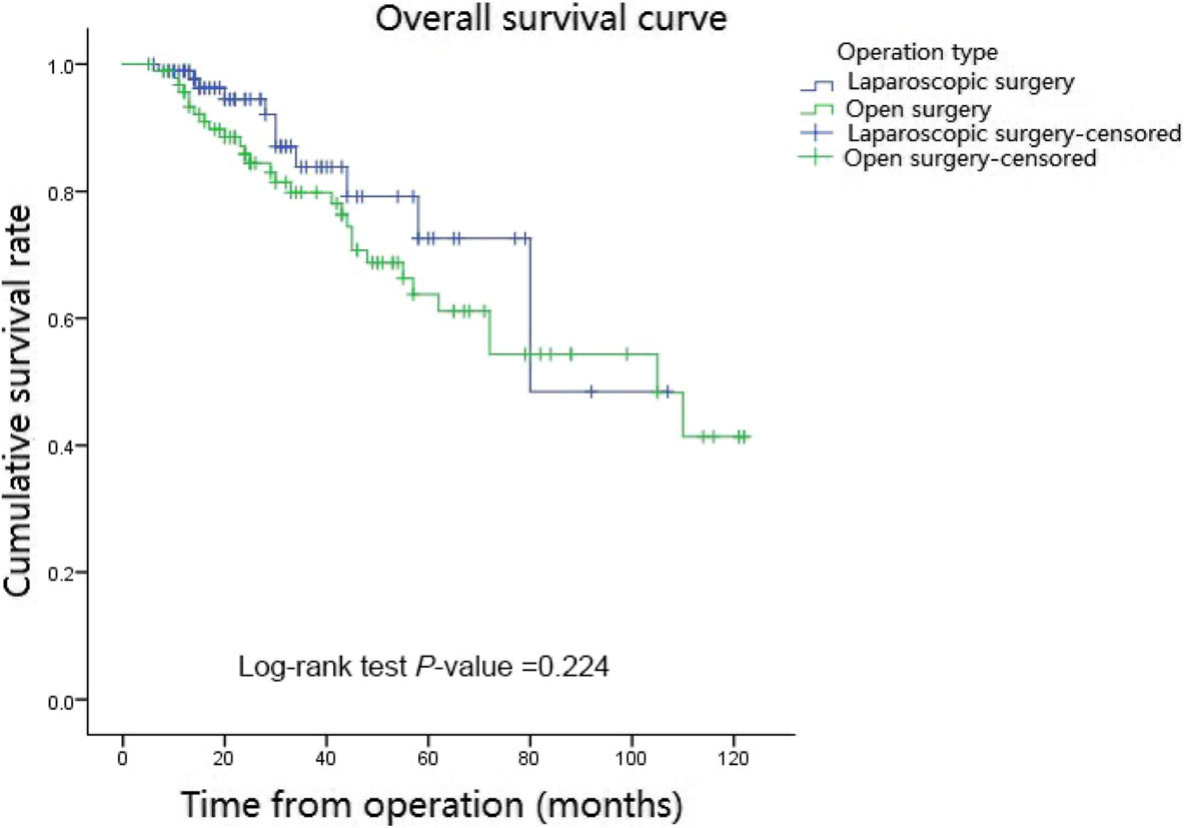
Overall Survival curve in matched cohort of laparoscopic and open groups. In the matched cohort, in the laparoscopic group, 3- year and 5-year Overall survival rates were 83.9 and 73.1% respectively and they were 79.6 and 63.4% respectively in the open group. There was no significant difference between the laparoscopic and open groups (*P* = 0.224)

**Fig. 2:**
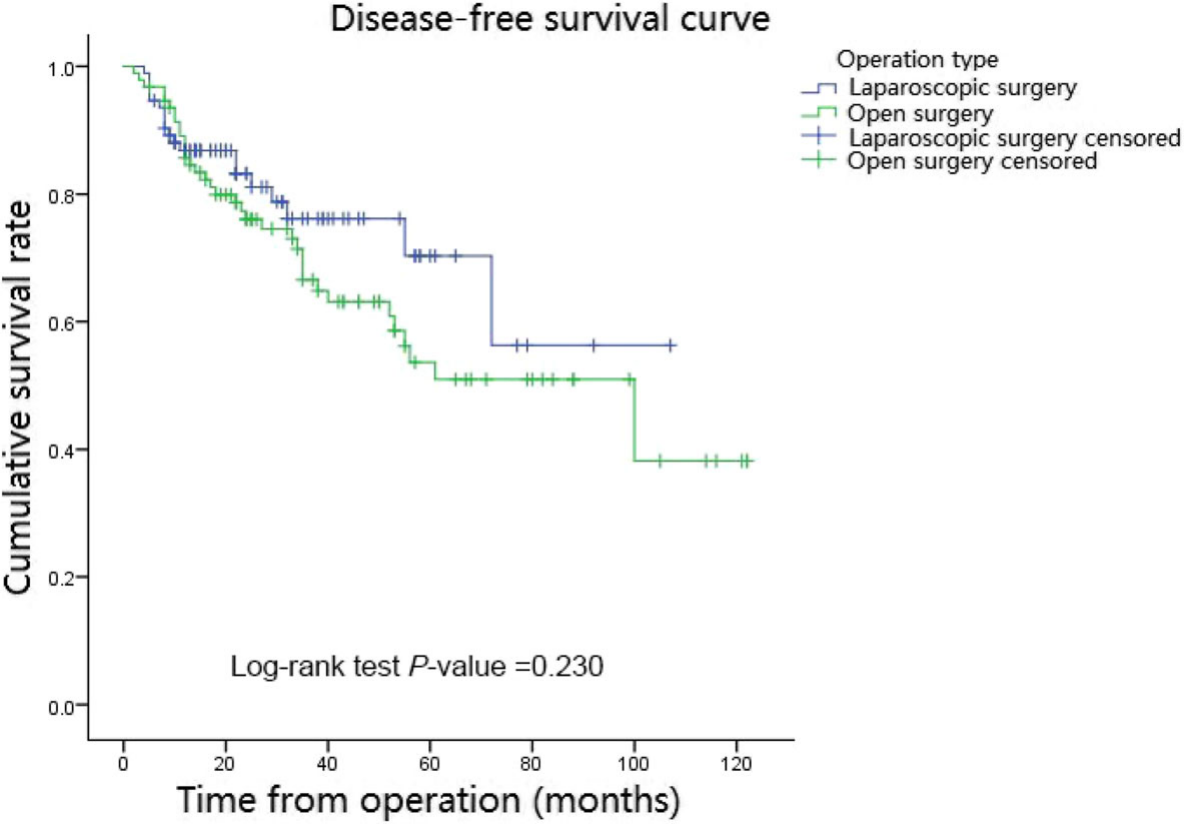
Disease-free curve in matched cohort of laparoscopic and open group. In the matched cohort, in the laparoscopic group, 3- year and 5-year disease-free survival rates were 76.3 and 69.9% respectively and they were 66.6 and 53.8% respectively in the open group. There was no significant difference between the laparoscopic and open groups (*P* = 0.230)

The mean follow-up period in the matched cohort was 37.4 months (range, 5–122 mouths; open group: 45.4 months; laparoscopic group: 29.5 months). During the whole follow-up period, 40 of the 186 patients died (21.5%) and 55 of the 186 patients had local recurrence or distant metastasis (29.5%). In the matched cohort, the Kaplan curves showed no statistically significant difference in OS (*P* = 0.224) and DFS (*P* = 0.230) between the two groups. Besides, the 3- and 5-year OS rates in the open group were 79.6 and 63.4% respectively and those in the laparoscopic group were 83.9 and 73.1%, respectively (Fig. 1). In addition, the 3-year DFS and 5-year DFS rates were 66.6 and 53.8% respectively in the open group, and they were 76.3 and 69.9% respectively in the laparoscopic group (Fig. 2).

At univariate analysis, CEA level, N stage, TNM stage, perineural invasion, and vascular invasion significantly affected both OS and DFS (*P* < 0.05). According to multivariate analysis, the OS was significantly affected by CEA level (HR: 2.32; 95% CI, 1.26–4.98; *P* = 0.022), TNM stage (HR: 9.82; 95% CI, 3.15–83.55; *P* = 0.002) and perineural invasion (HR: 1.57; 95% CI: 1.15–3.21; *P* = 0.041). Besides, DFS was significantly affected by the CEA level (HR:1.77; 95% CI:1.29–4.15; *P* = 0.038), TNM stage (HR:9.67; 95% CI:3.18–79.30; *P* = 0.012), and the perineural invasion (HR:2.09; 95% CI:1.59–5.32; *P* = 0.020) (Table 3).

**Table 3:** Univariate and multivariate analysis for overall survival and disease-free survival in matched cohorts

Variables	Overall survival				Disease-free survival			
	Univariate analysis		Multivariate analysis		Univariate analysis		Multivariate analysis	
	HR (95%CI)	*P*	HR (95%CI)	*P*	HR (95%CI)	*P*	HR (95%CI)	*P*
Gender: male/female	1.11 (0.60–2.08)	0.739			0.72 (0.42–1.23)	0.225		
Operative type: open/lap.*	1.30 (0.76–2.15)	0.224			1.41 (0.80–2.47)	0.230		
CEA > 5	1.53 (1.11–3.58)	0.008	2.32 (1.26–4.98)	0.022	2.35 (1.85–4.39)	< 0.001	1.77 (1.29–4.15)	0.038
ASA: I-II/III-IV	0.93 (0.73–1.94)	0.811			1.25 (0.64–2.13)	0.410		
Comorbidity	1.32 (0.78–2.33)	0.222			1.06 (0.53–1.51)	0.639		
Tumor size ≥5 cm	1.49 (0.97–2.95)	0.156			1.14 (0.63–1.97)	0.443		
N stage								
N0	Reference	–	Reference	–	Reference	–	Reference	–
N1	3.60 (1.56–8.30)	0.003	0.36 (0.05–2.77)	0.324	3.93 (1.94–7.96)	< 0.001	0.45 (0.06–3.46)	0.444
N2	14.37 (6.11–33.82)	< 0.001	1.03 (0.16–1.59)	0.854	12.25 (5.91–25.43)	< 0.001	1.46 (0.20–2.30)	0.714
TNM stage: I-II/III-IV	6.43 (2.93–14.10)	< 0.001	9.82 (3.15–83.55)	0.002	6.26 (3.22–12.18)	< 0.001	9.67 (3.18–79.30)	0.012
Differentiation	0.61 (0.31–1.21)	0.157						
Poor	Reference	–			Reference	–	Reference	–
Median	0.63 (0.32–1.25)	0.189			0.80 (0.52–1.47)	0.286		
High	0.72 (0.40–1.53)	0.279			0.75 (0.45–1.33)	0.248		
Perineural invasion	4.24 (2.21–8.14)	< 0.001	1.57 (1.15–3.21)	0.041	2.12 (1.42–4.25)	0.002	2.09 (1.59–5.32)	0.020
Vascular invasion	2.72 (1.44–5.14)	0.002	1.23 (0.61–2.47)	0.075	1.78 (1.02–3.09)	0.041	1.63 (0.88–2.95)	0.133
Retrieved lymph node	0.85 (0.71–1.72)	0.533			0.79 (0.51–1.43)	0.273		
Postoperative complication	0.96 (0.84–1.98)	0.857			1.32 (0.70–2.41)	0.396		

*lap laparoscopic

## Discussion

According to this study, in patients older than 80 years old with colorectal cancer, laparoscopic surgery has better short-term outcomes than the open surgery but there is no significant difference for the long-term survival outcomes, CEA level, III/IV stage, and perineural invasion were all reliable predictor of overall survival and disease-free survival for either laparoscopic or open surgery.

Previous studies had already shown that elderly colorectal cancer patients could also obtain better short-term outcomes through laparoscopic surgery [10, 14–19]. In the current study, it was found that the laparoscopic surgery could significantly reduce the intraoperative blood loss and postoperative complication. According to previous report, reduction of blood loss could reduce the stress reaction of surgery and further greatly reduce the incidence of postoperative complications, hence, the reduction of blood loss could effectively improve the postoperative recovery of patients [20]. Besides, among the postoperative complication, the laparoscopic surgery could significantly decrease the incidence of grade I-II complication such as wound infection compared to the open surgery. Moreover, laparoscopic surgery could significantly increase the number of the retrieved lymph node. This was possibly attributed to clear and magnified visualization under laparoscopy, and was consistent with the report of Yang et al. which showed that the laparoscopic could significantly increase the number of retrieved lymph nodes for the early distal gastric cancer [12]. Previous studies had revealed the advantages of laparoscopic surgery about the faster recovery [21–24]. Vignali et al. had reported laparoscopic surgery could significantly decrease the time to first flatus, the time to liquid diet, and hospital stay [23]. Consistent with the above reports, the current study found that compared with the open group, the laparoscopic surgery could significantly reduce the time to flatus, time to oral feeding, and postoperative hospital stay. Overall, the above findings reflected that the laparoscopic surgery had better short-term outcomes in the treatment of elderly patients with colorectal cancer than the open surgery.

Few studies reported data regarding long-term outcomes of laparoscopic surgery [25, 26]. In 2015, Hinoi et al. reported that there was no significant difference for octogenarian patients with rectal or colon cancer in 3-year overall survival, disease-free survival, and cancer-specific survival between laparoscopic and open groups [26]. Likewise, in 2016, Moon et al. reported that the laparoscopic surgery was without any significant difference for the 3- and 5-year overall survival, and 3-year and 5-year recurrence-free survival compared to the open surgery [25]. In this study, no difference in the 3-year and 5-year OS rates (*P* = 0.224) and in 3- year and 5-year DFS rates (*P* = 0.230) were observed between the open and laparoscopic surgery. Besides, it is noteworthy that the 3-year and 5-year OS rates, and 3- year and 5-year DFS rates of patients in the laparoscopic group were generally higher than the open group. The 5-year DFS rate in the laparoscopic group was even higher than that in the open group by more than 10%. This difference might be due to the difference in the number of dissected lymph node between the open group and the laparoscopic group. Hence, although there was no significant difference in survival outcomes between the two surgical methods, the laparoscopic surgery in elderly patients with colorectal cancer might achieve better survival outcomes than the open surgery.

Prognostic factors affecting the survival of colorectal cancer patients have been previously reported [27–30]. Huh et al. had reported that both preoperative CEA level, TNM stage, and vascular or neural invasion were independent prognostic factors for the overall survival and disease-free survival in potentially curative colorectal cancer [30]. Besides, Tsai et al. reported the perineural invasion as a significant prognostic factor for postoperative relapse for stage II colorectal cancer undergoing radical resection [27]. Consistently with the previous studies, in this study, it was found that CEA level, III/IV stage, and perineural invasion were all independent predictors for the overall survival and the disease-free survival of elderly patients with colorectal cancer.

This study has the limitations of any retrospective study. However, selection bias was reduced by propensity score matching through logistic regression. Multicenter large-scale prospective studies are needed to further confirm whether laparoscopic treatment is more suitable for elderly patients with colorectal cancer in terms of short-term and survival outcomes. Cutoff values for CEA level, III/IV stage, and perineural invasion were not evaluated in this study, so large-scale studies are necessary to determine specific valid cutoff values for CEA level, III/IV stage, and perineural invasion.

## Conclusions

Laparoscopic surgery showed better results than the open surgery in short-term outcomes. CEA level, III/IV stage, and perineural invasion were all reliable predictor of overall survival and disease-free survival for the treatment of laparoscopic surgery and open surgery for elderly Chinese patients over 80 years old with colorectal cancer.

## Data Availability

The datasets generated and/or analysed during the current study are not publicly available due to the data is confidential patient data but are available from the corresponding author on reasonable request.

## Abbreviations

ASA: American Society of Anesthesiologists
BMI: body mass index
CEA: carcinoembryonic antigen
CI: confidence interval
CRM: circumferential resection margin
DFS: disease-free survival
HGB: hemoglobin
HR: hazard ratio
ICU: intensive care unit
OS: on overall survival
PSM: Propensity score matching
TME: total mesorectal excision
TNM: tumor nodes metastasis
